# Thioredoxin-Interacting Protein (TXNIP) with Focus on Brain and Neurodegenerative Diseases

**DOI:** 10.3390/ijms21249357

**Published:** 2020-12-08

**Authors:** Haruka Tsubaki, Ikuo Tooyama, Douglas Gordon Walker

**Affiliations:** Molecular Neuroscience Research Center, Shiga University of Medical Science, Seta-Tsukinowa, Otsu 525-0072, Shiga, Japan; ds111857@g.shiga-med.ac.jp (H.T.); kinchan@belle.shiga-med.ac.jp (I.T.)

**Keywords:** oxidative stress, inflammation, Alzheimer’s disease, glucose metabolism, neuropathology

## Abstract

The development of new therapeutic approaches to diseases relies on the identification of key molecular targets involved in amplifying disease processes. One such molecule is thioredoxin-interacting protein (TXNIP), also designated thioredoxin-binding protein-2 (TBP-2), a member of the α-arrestin family of proteins and a central regulator of glucose and lipid metabolism, involved in diabetes-associated vascular endothelial dysfunction and inflammation. TXNIP sequesters reduced thioredoxin (TRX), inhibiting its function, resulting in increased oxidative stress. Many different cellular stress factors regulate TXNIP expression, including high glucose, endoplasmic reticulum stress, free radicals, hypoxia, nitric oxide, insulin, and adenosine-containing molecules. TXNIP is also directly involved in inflammatory activation through its interaction with the nucleotide-binding domain, leucine-rich-containing family, and pyrin domain-containing-3 (NLRP3) inflammasome complex. Neurodegenerative diseases such as Alzheimer’s disease have significant pathologies associated with increased oxidative stress, inflammation, and vascular dysfunctions. In addition, as dysfunctions in glucose and cellular metabolism have been associated with such brain diseases, a role for TXNIP in neurodegeneration has actively been investigated. In this review, we will focus on the current state of the understanding of possible normal and pathological functions of TXNIP in the central nervous system from studies of in vitro neural cells and the brains of humans and experimental animals with reference to other studies. As TXNIP can be expressed by neurons, microglia, astrocytes, and endothelial cells, a complex pattern of regulation and function in the brain is suggested. We will examine data suggesting TXNIP as a therapeutic target for neurodegenerative diseases where further research is needed.

## 1. Introduction

Oxidative stress resulting from an imbalance in cellular redox can occur due to the production of excessive levels of reactive oxygen species (ROS) and/or a deficit of cellular antioxidant systems. Supplementation with various classes of antioxidants has been widely investigated for neurodegenerative diseases (for recent reviews, see [1,2]). Excess levels of ROS can occur from inflammatory responses by innate immune cells (e.g., neutrophils, monocytes/macrophages/microglia) due to infections or chronic autoimmune responses [2], deficiencies in mitochondrial oxidative phosphorylation pathways [3,4], and environmental factors such as UV light, toxic chemicals, or heavy metals. Oxidative stress is linked to many different chronic human diseases, particularly those with inflammatory and/or metabolic components (e.g., diabetes and diabetes-related retinopathy [5], stroke [6], cancers [7], vascular diseases, and neurodegenerative diseases [8,9]). Developing effective therapies for these diseases requires the identification of key pathological targets responsible for amplifying disease processes.

Thioredoxin-interacting protein (TXNIP) has emerged as a key pathological regulator of diseases, particularly those associated with glucose and lipid abnormalities and inflammation. TXNIP has been identified in disease mechanisms involved in various cancers [10,11,12], diabetes mellitus [13], cardiovascular disease [14], renal disease [15], and retinal disease [16], among others. Many of these diseases associated with TXNIP can arise from vascular complications of diabetes [17]. The involvement of TXNIP in diabetes, pancreatic beta-cell death, and glucose metabolism has been the focus of several recent review articles [14,17,18]. TXNIP was identified (and named) due to its interaction and inhibition of the key antioxidant proteins thioredoxin-1 (TRX1) and thioredoxin-2 (TRX2) [19,20,21], but more recent findings identified properties involved in cellular metabolism and transcription regulation [22], apoptosis and cell death [13], inflammation [23], and tumor suppression [11,24] that might not be directly related to the modulation of TRX and oxidative stress.

In this review, we will consider the known features of TXNIP expression with a particular focus on human and animal brains and different types of neural cells. Particular consideration will be on how TXNIP and the TRX system may be involved in the pathological features of Alzheimer’s disease (AD), a chronic neurodegenerative disease of elderly people, which have been associated with abnormalities in cellular glucose metabolism, mitochondrial dysfunction, inflammation, and oxidative stress, among others [25]. TXNIP might be an important hub coordinator of different pathological processes in this disease, but there has been only one published study on its expression and distribution in human AD brains, and further studies are needed [26]. We will also consider the evidence for the pathological contribution of diabetes and diabetes-induced oxidative stress to Alzheimer’s disease (AD) as part of this review [27], and how it can be linked to TXNIP.

## 2. Biochemistry of TXNIP: Overview

The gene for TXNIP is located on the human chromosome 1q21.1 and is transcribed to four messenger RNA (mRNA) splice variants [28]. TXNIP is a member of the α-arrestin protein family and has two arrestin-like domains: one is a PxxP sequence and the other is a PPxY sequence. One mRNA for TXNIP codes for 336 amino acids for a polypeptide of approximately 37.4 kDa, whereas another codes for 391 amino acids for a polypeptide of approximately 43.7 kDa. The two other splice variants have not been associated with proteins. Reported molecular weights of TXNIP vary depending on the expressing cell type, but the major protein band(s) detected by SDS-gel electrophoresis is/are 50–55 kDa. This would suggest a certain amount of post-translational modification occurring. Protein bands of approximately 37 kDa have been observed on immunoblots with certain antibodies and cell types, but 50–55 kDa polypeptides appear to be the major form. TXNIP was identified initially by several investigators as vitamin D3-upregulated protein-1 (VDUP1) in different cell types [19,20,21]. Interestingly, other studies have not confirmed TXNIP induction by vitamin D3 in additional cell types, and noticeably, there is a lack of a vitamin D3-responsive element in the TXNIP gene promoter [29,30]. It has been suggested that vitamin D3 stabilizes cellular TXNIP protein rather than inducing expression [29].

### 2.1. TXNIP and Thioredoxin System

Earlier studies provided the first link between VDUP1/TXNIP and oxidative stress by demonstrating binding to reduced, but not oxidized, thioredoxin (TRX) [19,20,21]. TXNIP can bind two cysteine residues (Cys32 and Cys35) present in the active catalytic site of TRX. The binding of oxidized TXNIP to reduced TRX involves the formation of disulfide bonds between TXNIP-Cys247 and TRX-Cys32 [31]. A mutation in TXNIP-Cys247 is sufficient to remove its ability to sequester TRX activity [31]. Although classified as belonging to the α-arrestin family, these cysteine residues are unique to TXNIP. TXNIP can bind and inactivate both TRX1 and TRX2 [19,32]. The TRX cellular disulfide oxidoreductase system is a highly conserved system found from prokaryotes to plants to mammals [33,34,35]. TRX plays a central role in protecting cells from oxidative stress. Oxidized TRX is reduced by NADPH through a reaction catalyzed by thioredoxin reductase complex (TRX-R). Reduced TRX can then directly reduce disulfides in target proteins [33]. The main form of TRX is TRX1, which is located primarily in the cytosol but can be translocated to the plasma membrane and/or nucleus, particularly under inflammatory conditions [36,37]. Closely related in structure, TRX2 is specific to mitochondria [38,39]. Immunohistochemistry and in situ hybridization mRNA studies have shown that TRX was highly expressed in mammalian brains, particularly in subsets of neurons in areas of high metabolic activity and oxidative burden [40,41,42]. TRX mRNA expression was highest in the piliform cortex, dentate gyrus, CA3/CA4 region of the hippocampus, locus coeruleus, and nucleus of the hypothalamus and solitary tract [41]. Trx2 mRNA and protein were highly expressed in rat brains in neurons in the olfactory bulb, frontal cortex, hippocampus, some hypothalamic and thalamic nuclei, cerebellum, and numerous brainstem nuclei [42].

The significance of the TRX system for maintaining cellular health was demonstrated in transgenic mice overexpressing forms of TRX. Increased expression of human TRX in mice resulted in resistance to oxidative stress and increased lifespan [43]. Other studies have shown that TRX overexpression in transgenic mice was protective of transient ischemic brain damage [44]. This effect was also observed by intravenous administration of TRX protein [45]. These examples of the beneficial outcomes of increased TRX are used to introduce the widely appreciated pathological consequences of reduced TRX activity. The first study linking TRX to AD showed a significant reduction in TRX protein levels in most brain regions in AD cases compared to nondemented controls with accompanying increased TRX-R activity. This study also demonstrated the protective effect of TRX when added to cultures of neurons treated with toxic doses of amyloid beta (Aβ) peptide [46]. A further study showed that TRX1 levels were increased significantly in the cerebrospinal fluid (CSF) and plasma of AD cases compared to mild cognitive impairment (MCI) cases [47]. This suggested that TRX1 was being secreted from damaged neurons in AD brains. A noticeable alteration in the patterns of TRX1 and TRX2 cellular immunoreactivity in hippocampal neurons of AD cases was also observed.

### 2.2. TXNIP Interacting Proteins

TXNIP has been shown to interact with other proteins besides TRX, including importin-α1 [48], human ecdysoneless (hEcd) [49], and NOD-, LRR-, and pyrin domain-containing protein-3 (NLRP3), a component of the inflammasome complex [23,50]. These interactions relate to additional identified properties of TXNIP. The significance of the interaction of TXNIP with inflammasome components and enhancement of inflammation will be considered.

## 3. Regulation of TXNIP Expression

Many different cellular stress factors positively or negatively regulate TXNIP expression. These include UV light, heat shock [51], hypoxia, ROS, nitric oxide [52], nicotinamide adenine dinucleotide [53], ATP, glutamine, nicotine, vascular endothelial growth factor, basic fibroblast growth factor [54], transforming growth factor β [55], estradiol [56], calcium channel blockers [57], activators of advanced glycation endoproduct receptor (RAGE) [58], insulin, and glucose [59]. Activation of the TXNIP promoter, which contains a carbohydrate response element (ChoRE), is regulated by transcription factors MondoA:Max-like protein X (MLx), nuclear factor Y (NF-Y), and the carbohydrate response element-binding protein (ChREBP) [45]. Transcription factors forkhead box O1 (FOXO1) and FOXO3a can bind to the TXNIP promoter and by competing with ChREBP can downregulate TXNIP transcription [46,47]. Activation of AMP protein kinase (AMPK) can also lead to inhibition of TXNIP mRNA transcription [48].

The mechanism whereby TXNIP expression is induced through the activation of the receptor for advanced glycation endproducts (RAGE) by the RAGE ligand S100b is of relevance for th consideration of AD as RAGE is a receptor for Aβ and is involved in Aβ toxicity and AD pathogenesis [58]. It was demonstrated in Schwann cells in vitro and injured sciatic nerve in vivo that binding of S100b to RAGE induced TXNIP with TXNIP being involved in the downstream activation of p38 mitogen-activated protein kinase (MAPK), cAMP response element-binding protein (CREB), and nuclear factor κB (NFκB). RAGE silencing blocked the induction of TXNIP, whereas the silencing of TXNIP inhibited the activation of these signaling pathways, preventing RAGE-induced fibronectin and IL1β synthesis and Schwann cell migration [58]. Induction of TXNIP by RAGE ligands in retinal endothelial cells through the activation of p38 MAPK and NFκB also led to enhanced expression of inflammation-associated genes, including cyclooxygenase 2 (*Cox2*), vascular endothelial growth factor (*VEGFA*), and intercellular adhesion molecule-1 (*ICAM1*). Expression of these genes was reduced when TXNIP expression was inhibited, and enhanced with TXNIP overexpression [60].

### 3.1. Cell Types

TXNIP expression has been studied in a range of different cell types derived from different tissues. Expression of TXNIP is not tissue-specific, with TXNIP RNA being detected across all tissues and many different cell types. The highest expression levels were demonstrated in the lungs, thyroid glands, adipose tissue, skeletal muscle, and blood mononuclear cells (https://www.proteinatlas.org/ENSG00000265972-TXNIP/summary/rna). By comparison, expression in neural tissue was relatively low, with the highest levels in the retina. The highest levels of constitutive expression of TXNIP mRNA were in muscle-derived cell lines (https://www.proteinatlas.org/ENSG00000265972-TXNIP/summary/rna).

A common feature of all expressing cell types has been the induction of TXNIP by different forms of cellular stress. Increased levels of palmitate and other lipids that led to cellular endoplasmic reticulum (ER) stress [61,62], increased levels of N-methyl D-aspartate (NMDA) resulting in NMDA receptor activation, and increased cellular calcium [63], increased ischemia/hypoxia, and increased glucose, resulting in enhanced oxidative stress, which all induced TXNIP expression. Increased levels of ROS from dysfunctional mitochondria can also increase TXNIP expression [32].

The promoter for VDUP1/TXNIP contains many different cis-regulatory sequences, but the presence of the heat shock factor element (HSE) sequence appeared critical for controlling cellular responses to certain stresses [51]. Using HEK-293 human embryonic kidney-derived Bosc cells, TXNIP was upregulated when cells were grown at high density or with serum deprivation, but this effect was abolished if the HSE sequence was deleted from the promoter. Overexpression of heat shock factor (HSF) in these cells upregulated the expression of TXNIP [51]. Many different stress factors have been shown to induce TXNIP expression in different cell types. One common factor can be ER stress. In an experimental cell model using INS-1 rat islet-derived cells, the induction of unfolded protein response (UPR) due to thapsigargin-induced ER stress resulted in the rapid induction of TXNIP expression along with the UPR sensor molecules Inositol-requiring transmembrane kinase/endoribonuclease 1α (IRE1α) and protein kinase RNA-like ER kinase (PERK). In cells deficient in IRE1α and PERK, an ER stress-induced increase in TXNIP did not occur. Activated IRE1α increased TXNIP mRNA stability by reducing levels of a TXNIP-destabilizing microRNA, miR-17. As a result of elevated TXNIP protein expression, NLRP3 becomes activated, resulting in the formation of caspase-1 and bioactive IL-1β [50]. This mechanism is believed to be a key event in diabetes, as excess TXNIP leads to pancreatic cell death, whereas knockdown of TXNIP prevented ER stress-induced cell death [64]. It has been previously shown that excess glucose-induced TXNIP expression by activating the transcription factor carbohydrate response element-binding protein (ChREBP) causes binding to an element in the TXNIP promoter and increasing transcription [65]. Activation of ChREBP and translocation to the nucleus also occurred as a result of ER stress [64]. The described mechanism for the ER stress-induced unfolded protein response (UPR) could have direct relevance for neurodegenerative diseases such as AD and Parkinson’s disease (PD) where accumulations of toxic Aβ or α-synuclein, primarily produced by neurons, can induce UPR [66,67]. Other transcription factors can positively or negatively affect the expression of TXNIP. Overexpression of transcription factor Ets-1, which is activated by inflammatory/oxidative stress associated extracellular regulated kinase (ERK1/2), resulted in increased TXNIP in Min6 β-cells and mouse islet cells. Knockdown of Ets1 reduced the expression of TXNIP. Overexpression of Ets and TXNIP negatively affected cellular proliferation and glucose regulatory functions [68].

#### 3.1.1. Peripheral Cells

Many studies of TXNIP cellular expression in peripheral cells have focused on pancreatic islet cells in terms of effects on glucose uptake and metabolism, insulin secretion, and cell death. These studies have been extensively reviewed for their significance in understanding mechanisms of diabetes (for recent reviews, see [69,70]). For this review focusing on brain diseases with inflammatory components, we will consider TXNIP expression in macrophages and its consequences on inflammation. Increasing TXNIP expression in human blood-derived macrophages with a peroxisome proliferator-activated receptor-gamma (PPAR-γ) agonist resulted in enhanced levels of activated caspase-3, indicative of inflammasome activation, and also increased apoptosis [71]. In contrast, stimulating macrophages with a PPAR-alpha agonist resulted in increased TRX and reduced the expression of TXNIP, reduced caspase-3 activation, and reduced numbers of apoptotic cells [72]. Induction of TXNIP in RAW264 murine macrophage cells with the sugar D-allose or by plasmid-mediated overexpression inhibited the differentiation of osteoclasts by inhibiting the transcriptional activity of activator protein 1, NFκB, and nuclear factor of activated T cells (NFAT) [73]. Earlier studies have identified D-allose to have significant antiproliferative properties in several types of cancer cells [74,75,76] through the activation of the transcription factor MondoA:Mlx and the induction of TXNIP expression [77].

TXNIP deficiency due to treatment with lipopolysaccharide (LPS) used as a model of endotoxic shock can exacerbate inflammation. Macrophages from TXNIP-deficient mice produced increased amounts of nitric oxide in response to LPS treatment, and in vivo, TXNIP-deficient mice were rescued from endotoxic-shock-induced death by a nitric oxide synthase inhibitor. The mechanism is due to the LPS inhibition of MondoA:Mlx activation preventing the induction of TXNIP expression [78,79]. In contrast to LPS, chemicals that induce endoplasmic reticulum (ER) stress in macrophages, including tunicamycin, produced enhanced inflammation through increased production of ROS causing TXNIP induction NFκB activation and inflammasome/caspase-1 activation resulting in enhanced IL-1β secretion [80].

#### 3.1.2. Neurons

The first study describing TXNIP function in neural cells employed cerebellar granular neurons, which underwent apoptosis when culture conditions were switched from high potassium depolarizing conditions to physiological potassium conditions. TXNIP expression was significantly enhanced in neurons under this and other apoptosis-inducing conditions. TXNIP induction was inhibited by increased calcium influx through voltage-dependent calcium channels [57]. Glutamate neurotoxicity was shown to be dependent on TXNIP inflammasome activation. Treatment of mice hippocampal slice cultures or SH-SY5Y cells with toxic doses of glutamate resulted in increased ROS, ER stress, increased TXNIP expression, and NLRP3 inflammasome activation, resulting in cell toxicity [81]. Treatment with the antioxidant curcumin was protective by the activation of AMPK and reducing the activation of IRE1α and PERK, resulting in the decreased expression of TXNIP, reduced NLRP3 inflammasome activation, and reduced cell death. Curcumin increased AMPK activity, whereas knockdown of AMPKα with specific siRNA abrogated its inhibitory effects. The protective effects of curcumin on inhibiting phosphorylated (p)-IRE1α, p-PERK, and NLRP3 expression in the hippocampus CA1 region was also demonstrated in vivo in rats subjected to hypoxia/ischemia insults [81]. A recent study that characterized the gene expression profiling of SH-SY5Y-derived neurons stably overexpressing the mitochondria-localized antioxidant protein mitochondrial ferritin (FtMt) demonstrated significantly reduced constitutive levels of expression of TXNIP mRNA. As increased FtMt will reduce ROS produced by mitochondria, this finding demonstrated a link between mitochondria and TXNIP expression in neurons [82]. Over-expression of FtMt and reduced TXNIP was protective for these cells.

#### 3.1.3. Microglia

The interaction of ROS/oxidative stress and inflammasome activation can be demonstrated in microglia, the major mediator of innate inflammatory responses in the brain. Microglia treated with thrombin, a factor involved in brain injury after infarct or hemorrhage, expressed increased amounts of ROS and TXNIP, causing inflammasome activation and apoptosis. Treatment with N-acetyl cysteine, a free-radical scavenger, prevented TXNIP induction, resulting in cell death [83]. Resveratrol, a polyphenol with antioxidant, sirtuin, and PPAR-γ-activating properties, inhibited Aβ-induced inflammatory activation and proliferation of microglia by inhibiting the activation of TXNIP/TRX/NLRP3 inflammasome pathway [84]. As a model of chronic stress, treatment of primary mouse microglia with the stress hormone corticosterone increased TXNIP expression, TXNIP/NLRP3 interactions, and IL-1β levels, indicating inflammatory activation [85]. Knocking-down TXNIP expression significantly inhibited corticosterone-induced inflammatory activation. Similar findings were shown in mouse brains of corticosterone-treated animals [85].

A recent RNA sequencing profiling study of microglia purified from AD model mice identified the decreased expression of TXNIP between microglia considered homeostatic and microglia activated to a stage 1 disease-associated microglia (DAM) [86,87]. Similarly, a number of homeostatic microglial genes (*P2ry12*, *P2ry13*, *Tmem119*, *Cxcr1*) were also downregulated between these groups. DAM is a newly defined phenotype describing activated plaque-associated microglia with the function to reduce the amplification of inflammation.

#### 3.1.4. Astrocytes

Astrocytes, the major support cells of the central nervous system providing growth factors, metabolic support, and detoxifying potentially damaging molecules (e.g., glutamate), can become activated by inflammatory cytokines and contribute to neurodegenerative processes by increased ROS and nitric oxide production. In a rat model of obesity, high-fat diet (HFD)-fed rats, both wild-type and spontaneously hypertensive rats, had evidence of abnormal capillary formation and degeneration along with the enhanced expression of TXNIP, NFκB, and inflammatory cytokines IL-1β and TNFα [88]. Increased expression of TXNIP was detected in astrocytes, retinal endothelial cells, and some microglia. Increased interaction of TXNIP and NLRP3 was also demonstrated in brain tissues from HFD rats. In a model of chronic mild stress/depression, deletion of the mitochondrial uncoupling protein-2 resulted in enhanced oxidative stress, enhanced TXNIP expression, and NLRP3 inflammasome activation in astrocytes [89]. In a rat model of subarachnoid hemorrhage, the peak induction of TXNIP correlated with the peak of cell death. Inhibition of TXNIP expression and the use of ER stress inhibitors resulted in an insignificant reduction of cell death. TXNIP expression was detected in astrocytes, microglia, and neurons in this model [90]. The role of astrocyte TXNIP in cellular lactate and glucose energy metabolism was demonstrated [91]. Activation of AMPK in astrocytes inhibited TXNIP by its phosphorylation and by promoting its degradation and inhibition of transcription. Activation of AMPK resulted in improved levels of glut-1 transporter expression in astrocytes. In AMPK astrocyte-specific deletion mice, glucose and lactate metabolism was impaired by high TXNIP expression resulting in an energy imbalance in neurons and, consequently, and cell death [91].

#### 3.1.5. Endothelial Cells

TXNIP is involved in vascular dysfunction due to damage to vessel endothelial cells, the major cause of complications resulting from diabetes [92]. A number of studies have shown common mechanisms that high glucose and high levels of oxidized low-density lipoproteins (oxLDL) result in oxidative stress and inflammation through the enhanced expression of TXNIP and inflammasome activation. As an example, treatment of endothelial cells with high glucose and oxLDL combined with rosmarinic acid, an anti-inflammatory and antioxidant, inhibited TXNIP expression, inflammasome activation, and endothelial dysfunction through the inhibition of p38 MAPK and FOXO1 signaling. Similar results were obtained with ROS scavengers and p38 MAPK and FOXO1 inhibitors [93]. TXNIP knockout mice were shown to be resistant to the retinal endothelial dysfunction that occurred with a HFD in wild-type mice. The TXNIP gene deletion mice did not have the breakdown of the blood–retinal endothelial barrier that occurred in 18-week HFD-fed wild-type mice [94]. Overexpression of TXNIP in human aortic endothelial cells induced by high glucose or the overexpression of ChREBP directly caused apoptosis and impaired NO function. In vivo, type-1 diabetes modeled in rats caused increased TXNIP in peripheral blood along with decreased NO and VEGF, increased ROS, and increased inflammation-associated vascular cell adhesion molecule 1 (VCAM-1) [95].

#### 3.1.6. Retinal Pigment Epithelial Cells

Retinal pigment epithelial (RPE) cells are the major support cells for the photoreceptor neurons in the eye. These cells transport nutrients, ions, and water, protect against photooxidation, reisomerization of all-trans-retinal into 11-cis-retinal, crucial for the visual cycle, phagocytosis of damaged photoreceptor constituents, and secrete essential factors for the structural integrity of the retina, for example pigment epithelial-derived factor (PEDF). These cells are damaged by the high-glucose/oxidative stress consequences of diabetes [96], and their dysfunction is involved in the development of diabetic retinopathy [97]. High-glucose-induced TXNIP in RPE cells resulted in mitochondrial depolarization, mitochondrial fragmentation, and shuttling to lysosomes. TXNIP overexpressing RPE cells showed significant changes in lysosome structure and autophagy. These high-glucose effects were inhibited if TXNIP expression was suppressed [98]. Surprisingly, another study demonstrated that the downregulation of TXNIP by oxidative stress in the RPE cell line ARPE-19 resulted in reduced cellular proliferation. The consequences of downregulation of TXNIP were increased autophagic flux, and increased p53 activation resulting in AMPK activation. The consequences of these were reduced blood–retinal barrier integrity [99].

#### 3.1.7. Brain Cancer Cells

Overexpression of TXNIP has been correlated with inhibition of tumor cell suppression in multiple studies. In addition, alterations in the expression of all proteins of the TRX system, including TRX, TRX reductase (TRXR), and TXNIP, in gliomas and glioblastomas were associated with differing clinical outcomes. High cytoplasmic TRXR and TRX expression in patients with high-grade gliomas was associated with poor outcomes, whereas in medulloblastoma, high expression of cytoplasmic TRXR, TRX, and nuclear TRX was associated with worse prognosis [100]. In similar studies of malignant glioma, higher TXNIP expression levels were associated with extended patient survival time [24]. In vitro analysis in U251 cells showed increased growth, migration, and invasion with downregulated TXNIP expression compared with their non-transfected counterparts. Although not studied in brain cancer cells, it was shown in thyroid cancer cells that TXNIP overexpression resulted in decreased glucose uptake and cell division. In a mouse model, transplanted TXNIP-overexpressing cells had reduced metastatic potential [101]. A similar feature was observed in prostate cancer cells. Overexpression of TXNIP in a prostate cancer cell line blocked cell proliferation and glucose uptake. Cell division became halted in the G0/G1 phase, which promoted cell apoptosis [11]. This study also showed in a large series of human prostate benign hyperplasia and cancer samples that there was significantly less TXNIP expression in the cancer samples. Levels of TXNIP in these samples negatively correlated with the expression of glucose transporter (*glut1*)-1 mRNA [11].

## 4. TXNIP, Hyperglycemia, and Oxidative Stress—NLRP3 Inflammasome Complex

Presentations of research data on TXNIP in relation to disease have frequently considered TXNIP/TRX interactions and the resulting oxidative stress as a separate mechanism from its role in the NLRP3 inflammasome activation, but these two features are interrelated. Although associated with enhanced inflammation, NLRP3 inflammasome activation occurs in all brain cell types, including neurons, astrocytes, and endothelial cells, not just microglia [81,102]. If one considers the events occurring under hyperglycemic conditions, excess glucose leads to increased levels of ROS by inducing the overproduction of NADH and increased mitochondrial-derived ROS that inhibits GAPDH, the enzyme that removes excess cellular glucose [103]. However, this inhibition that activates alternative glucose metabolic pathways leads to further ROS production. Excess ROS directly induces the expression of TXNIP and the inflammation-associated transcription factor NFκB. Excess TXNIP will further exacerbate oxidative stress by binding TRX and, in cooperation with NFκB, bind and activate the inflammasome complex. The inflammasome complex consists of an association of NLRP3, apoptosis-associated speck-like protein containing a caspase-binding domain (ASC) and pro-caspase-1 [104,105]. In the presence of excess ROS, TXNIP interaction with TRX can be reversed, which then allows released TXNIP to bind and activate NLRP3 inflammasome, resulting in enhanced inflammation. Activation of NLRP3-inflammasome promotes the formation of activated caspase-1, which processes interleukin (IL)-1β, IL-18, and IL-33 into bioactive forms [106], and also induces cell death through pyroptosis [107]. An earlier study also demonstrated that inflammasome activators such as uric acid crystals induced the dissociation of TXNIP from TRX in the presence of ROS, allowing it to bind and activate the NLRP3 complex and enhance caspase activation [50].

## 5. TXNIP Expression in the Brain

Studies focusing on the cellular distribution of TXNIP in brain cells are limited. Detailed immunochemical characterization of TXNIP/VDUP1 by comparing its distribution in *Drosophila* and rat brains showed relatively conserved patterns of expression. The antibodies employed in this study identified this protein in both of these species. Constitutive expression in subsets of neurons and astrocytes were identified using double immunohistochemistry staining with appropriate markers. This study did not report the constitutive microglial expression of TXNIP but observed nuclear immunoreactivity under hyperglycemic conditions [108]. A further study employed immunohistochemistry to compare the neuroanatomical distribution of different TRXs, TRRs, glutathione/glutaredoxin, peroxiredoxins, and TXNIP in rat brains. Expression of these antioxidants and related molecules was highest in brain regions susceptible to damage in conditions of hypoxia/ischemia including the cerebellum, cortex, hippocampus, substantia nigra, striatum, and spinal cord. Weak expression of TXNIP was detected in subsets of neurons, not glia, in these brain regions, except the spinal cord, and with the highest expression in the retina. The pattern of expression of TRX1 and TXNIP showed extensive overlap [109]. A direct link between metabolic activity and TXNIP was demonstrated in mice with the expression of TXNIP in medial hypothalamic neurons, which increased under conditions of nutrient excess and obesity [110]. A further study using directed TXNIP gene deletion and overexpression in *Agrp* hypothalamic neurons showed that the overexpression of TXNIP led to reduced energy expenditure and activity, resulting in obesity and fat accumulations, whereas the deletion of TXNIP in *Agrp* neurons had a reverse effect, with increased energy metabolism and activity [111]. Another study showed strong induction of TXNIP in the hypothalamus of mice in a state of lowered energy metabolism, though increased TXNIP expression was detected in ependymal-lining cells, not neurons, in the hypothalamus. Increased TXNIP expression was also detected in the liver and adipose tissues of these animals [112]. These studies highlighted physiological functions for TXNIP in regulating energy metabolism.

### 5.1. TXNIP in Acute Neurological Conditions

The role of TXNIP in ischemic stroke disease using animal models has been examined. Wild-type, TXNIP knockout, and wild-type mice treated with the TXNIP inhibitor resveratrol were subjected to middle cerebral artery occlusion (MCAO) to model ischemic stroke. These studies showed TXNIP knockout, and resveratrol-treated mice had an approximately 40% decrease in infarct size and significantly improved neurological scores. This improvement correlated with reduced oxidative stress and reduced evidence of NLRP3 inflammasome activation [113]. A similar model using rats subjected to MCAO with reperfusion was significantly protected by treatment with umbelliferone, an antioxidant. This protection occurred as treatment increased the expression of PPAR-γ, which resulted in reduced NLRP3 inflammasome activation due to the inhibition of TXNIP expression [114]. A recent study utilized neonatal rat pups subjected to common carotid artery ligand and hypoxia to induce ischemic injury [113]. Animals treated with a PPAR-β/δ agonist had a significantly reduced infarct area and improved neurological features. This study showed that PPAR-β/δ activation induced the expression of miRNA miR17-5p, which reduced TXNIP levels and the activation of Apoptosis signal-regulating kinase 1 (ASK1) and p38 MAPK [115]. Promoting TXNIP activation in vivo using a TXNIP CRISPR activation plasmid reversed the protective effects, as did the administration of a PPAR-β/δ antagonist. The consequence of PPAR-β/δ activation by GW0742 was decreased TXNIP, NLRP3, IL-6, and TNF-α and reduced numbers of activated microglia [116]. Similar results from PPAR activation were observed in vitro using PC12 neuronal-like cells subjected to oxygen/glucose deprivation (OGD) [116].

A recent study employing a rat model for cerebral venous sinus thrombosis demonstrated that neuronal pyroptosis was caused by endoplasmic reticulum stress and oxidative stress that activated the TXNIP/peroxynitrite/NLRP3 inflammasome pathway. Immunohistochemical staining revealed that increased TXNIP was primarily in neurons and also in subsets of microglia and astrocytes [117]. TXNIP immunoreactivity is colocalized with p-IREα1 and p-PERK, mainly in neurons. Time-course studies showed maximal induction of TXNIP occurred three days after surgery and then expression declined. The same time course of induction was observed for NLRP3, whose expression was in neurons and microglia. Direct interaction of TXNIP and NLRP3 at three days was demonstrated by immunoprecipitation and immunoblot analysis, producing results indicative of inflammasome activation [117], and at three days, IL-1β levels were also at their highest. A significant feature of this study was the decline of TXNIP and NLRP3 levels after reaching maximal levels. This suggests that the time for the increased expression of these proteins will be restricted and limited to ongoing active sites of pathology.

### 5.2. TXNIP in Neurodegenerative Diseases (Animal and Cellular Models)

**Figure 1:**
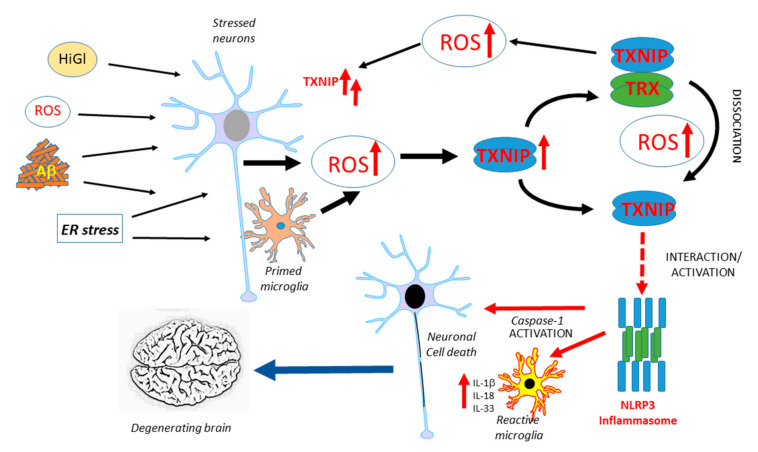
Overview of features of TXNIP expression and function that might contribute to AD pathogenesis. Multiple factors have been identified to be possible initiators of AD-related neurotoxicity, all of which have been shown to induce TXNIP. The potential interactions leading to enhanced inflammation and neurodegeneration are illustrated. ROS: reactive oxygen species, HiGl: High glucose, ER: endoplasmic reticulum, TXNIP: thioredoxin-interacting protein, TRX: thioredoxin, IL: interleukin.

TXNIP has been implicated in neurodegenerative diseases, but there have only been a limited number of studies on this topic. Figure 1 illustrates the potential interacting features of TXNIP in the progression of AD. Similar processes could occur in Parkinson’s disease (PD). This is a hypothetical model as further human-focused studies of brain expression of TXNIP are needed. Overexpression of α-synuclein, the protein implicated in the pathogenesis of PD, in transgenic mice or transfected HEK cells, produced increased levels of TXNIP [118]. In this study, it was also shown that the overexpression of TXNIP resulted in the enhanced accumulation of α-synuclein. This resulted in increased accumulation of the autophagy proteins LC3-II and p62, indicating excess TXNIP caused a blockade of autophagic flux. TXNIP impaired lysosomal function by downregulating the expression of the lysosomal membrane protein ATP13A2. Overexpression of TXNIP by the injection of a viral transduction vector into mice substantia nigra pars compacta resulted in a 38% decrease in the numbers of tyrosine-hydroxylase-positive neurons [118]. This was the first study linking TXNIP to PD pathogenesis, but there have been no studies on human brain tissues. The only study to date examining TXNIP expression in human AD and control brains observed an increased expression of TXNIP mRNA and protein in AD cases and increased TXNIP immunoreactivity of cells associated with Aβ plaques and phosphorylated tau (p-Tau)-positive structures. The identities of TXNIP-immunopositive cells were not confirmed but the authors implied that they were microglia due to their morphology as other components of the NLRP3 inflammasome complex and IL-1β levels were increased [26]. An experimental study using APP/PS1 plaque-developing transgenic mice demonstrated that TXNIP protein levels were significantly elevated whereas TRX levels were not in these mice at 9 and 12 months of age when Aβ deposits had developed compared to wild-type mice. The treatment of murine primary cortical neurons or HT22-derived hippocampal neurons with oligomeric Aβ (1-42) produced a significantly increased expression of TXNIP but not TRX [119]. Inhibiting TXNIP expression in Aβ-treated cells resulted in reduced amounts of oxidative modifications of cellular proteins. Wild-type mice treated with a regime of chronic unpredictable stress over 28 days had increased levels of TXNIP, but not TRX, protein in the frontal cortex and hippocampus. The consequence of this was evidenced by increased levels of cysteine oxidative modifications, such as increased protein sulfenylation and nitrosylation and increased amounts of phosphorylated (activated) ASK1 [120]. Another study showed increased levels of TXNIP and decreased levels of TRX in the hippocampus of the female 5xFAD line of amyloid plaque-developing mice. Using the SH-SY5Y neuroblastoma cell line, this study showed that estradiol could protect from TXNIP induction and cell toxicity induced by in vitro Aβ (1-42) treatment. The basis of this study was the analysis of human gene expression profiling data that showed significantly higher levels of TXNIP mRNA in samples from female AD compared to male AD subjects [121]. The effect of estradiol modulation of TXNIP upregulation was through the activation of AMPK. Using the 3xTg-AD mouse model, significantly enhanced TXNIP was detected in the cortex but coincident with significantly reduced expression in the spleen compared to wild-type mice. The mean spleen weight of 3xTg-AD mice at 17 months was three times that of wild-type mice, whereas levels of spleen TXNIP in 3xTg-AD mice was only approximately 5% of wild-type levels. The enhanced cerebrum TXNIP expression was associated with increased histone acetylation, increased transcription factor CCCTC-binding factor (CTCF) binding, and TXNIP promoter hypomethylation, whereas the reduced spleen TXNIP expression was associated with increased histone methylation, reduced CTCF binding, and TXNIP promoter hypermethylation. Increased cortical expression of TXNIP was detectable at seven months before increased levels of p-Tau and inflammatory mediators became significant. It was concluded that the interaction of the brain and peripheral TXNIP contributed to cognitive decline, but identifying mechanisms will require further study [122]. The relationship of TXNIP and enhanced tau phosphorylation was investigated in 5xFAD mice and SH-SY5Y neuroblastoma cells. Treatment of SH-SY5Y cells with Aβ (1-42) resulted in TXNIP overexpression, followed by enhanced oxidative stress, p38 MAPK, and increased tau phosphorylation at Ser202/Thr205. Knockdown of TXNIP significantly reduced TXNIP expression and tau phosphorylation in SH-SY5Y cells, whereas the treatment of 5xFAD mice with the voltage-dependent calcium channel blocker verapamil had similar effects [123]. A consensus on how TXNIP could be involved in AD-type degenerative processes is still lacking.

A separate series of studies identified features of NLRP3 inflammasome activation related to enhanced inflammation associated with AD pathology in human AD brains and mouse models [124,125,126,127]. These studies did not examine the involvement of TXNIP in the features of inflammasome activation, but it can be deduced based on other studies that it is involved in the observed pathological changes. It was shown that there was increased caspase-1 activation in AD and MCI human brains. In mice with genetic deficiencies of NLRP3, microglia showed a smaller inflammatory response and increased phagocytosis of amyloid beta [124]. It was shown in AD plaque-developing mice that injection of brain-derived ASC specks produced upon inflammasome activation promoted the seeding of Aβ plaques in brains [128].

Alterations in the expression of TXNIP have also been identified in blood cells from patients with multiple sclerosis (MS), a degenerative disease of white matter associated with autoimmune responses to myelin. In patients with newly diagnosed MS, for those receiving interferon or other immunosuppressive therapies, TXNIP mRNA expression was decreased whereas TRX1 mRNA was significantly increased. This suggested a disturbance in oxidative stress as part of the disease pathology [129].

### 5.3. Linking Diabetes, TXNIP, and Alzheimer’s Disease: Hypothesis

The role of TXNIP in inducing pancreatic cell death and causing type-1 diabetes appears to depend on properties not directly or exclusively related to its binding of TRX. A mutant form of TXNIP that does not bind TRX was shown to maintain glucose uptake inhibition properties, which was a property of the α-arrestin domain [130]. Subsequent studies identified that TXNIP inhibited the expression of glucose transporter-1 (Glut-1) and promoted endocytosis of the protein from the plasma membrane [131]. Activation of AMPK under conditions of energy stress phosphorylated TXNIP and promoted its degradation, preventing interactions with Glut-1 on the plasma membrane and restoring cellular glucose uptake and metabolism [131]. TXNIP also inhibits glucose uptake due to inhibiting glycolysis and oxidative phosphorylation. Cells lacking TXNIP showed increased glucose uptake [131]. How these features might be involved in AD is unclear but can be suggested based on glucose hypometabolism, a well-established feature of AD [132]. Glut-1 is highly expressed in the brain, particularly by cerebrovascular endothelial cells and astrocytes, whereas Glut-3 is the major neuronal transporter of glucose [133].

**Figure 2:**
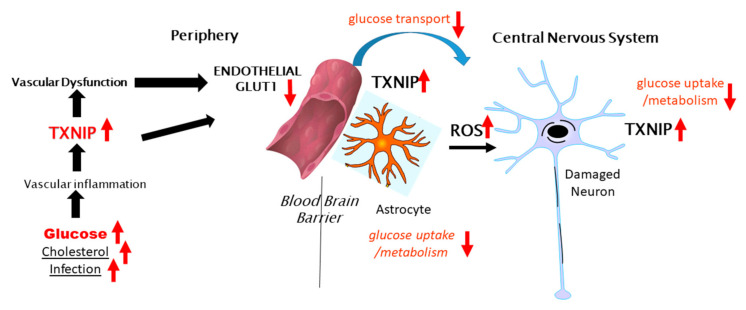
Potential peripheral–CNS interactions involving TXNIP in glucose metabolism in the brain.

Figure 2 illustrates the potential involvement of peripheral TXNIP in modulating cerebral glucose metabolism in the brain and AD, contributing to neurodegeneration. As several studies have shown that the neuronal expression of TXNIP is more commonly observed under stress/injury conditions, further experimental studies to determine if TXNIP overexpression affects Glut-3 expression and localization are needed. The concept of how AD neurodegeneration could be linked to diabetes, deficits in insulin signaling and glucose metabolism, and endothelial dysfunction should be considered. The term type-3 diabetes has been used to describe the lack of response of neurons to insulin and insulin-like growth factor signaling and the consequent deficits in glucose metabolism [134]. There have been epidemiological data suggesting patients with insulin-resistant type-2 diabetes are at greater risk of developing AD-type dementia and that antidiabetic medication was effective at reducing the risk of AD [135], though these findings have not been extensively confirmed. Examining the neuropathological records from an autopsy series, there was no significant correlation between type-2 diabetes and the severity of plaque and tangle load in brain regions affected by AD [136], with the exception of subjects with an apolipoprotein E4 allele. It was confirmed in a more recent study that type-2 diabetes did not correlate with the amyloid plaque or neurofibrillary tangle load, but it was commented on that both diseases are associated at early stages with a significant cerebrovascular disease that can affect the development of AD pathology [27]. Much interest in this topic has been on whether patients treated with the insulin sensitizer metformin have a reduced risk of dementia [137,138,139]. Metformin, which activates AMPK leading to inhibition of TXNIP expression, has significant activity in inhibiting the activation of the NLRP3 inflammasome complex [140,141,142]. Metformin also inhibited the interaction of TXNIP and NLRP3 [143]. Further studies are needed on human brains from cases with diabetes to determine whether there is increased expression of TXNIP and whether this is correlated with AD or severity of AD pathology.

## 6. Physiological Consequences of Loss of TXNIP

### 6.1. Experimental Animals

A number of studies have reported the phenotypes of mice constructed to have total TXNIP gene deletion. The consequences of this have been both beneficial and detrimental depending on the disease model. In an initial report, the cause of hyperlipidemia in a mutant mouse strain (Hcb-19) was a mutation in the *Txnip* gene resulting in reduced expression. These mutant mice had hypertriglyceridemia, hypercholesterolemia, elevated plasma apolipoprotein B, and increased secretion of triglyceride-rich lipoproteins with increased ketone body synthesis [144]. In a TXNIP gene-deleted mouse model, they developed significantly increased fatty liver, with high levels of triacylglycerol, cholesterol ester, and total cholesterol, with higher-serum non-esterified fatty acids. Elevated fatty acid synthesis was the primary cause of liver lipogenesis in TXNIP knockout mice [145]. Gene deletion of TXNIP also resulted in impaired immunity with reduced numbers of natural killer cells and the impaired dendritic cell maturation of T cells [146,147,148]. TXNIP gene deletion can lead to lethality under fasting conditions due to a switch in metabolism to hyperlipidemia and hypoglycemia. Under these conditions, mice experienced enhanced glucose-induced insulin sensitivity and secretion and an increased expression of PPAR-target genes [149]. TXNIP gene-deletion mice fed a high-fat diet developed significantly greater fat deposits and body mass but with increased insulin sensitivity. This study correlated the loss of TXNIP with the augmented activation of PPAR-γ [150]. These effects on enhanced hyperlipidemia due to TXNIP gene deletion were detrimental to the affected organisms, but beneficial outcomes upon reversing the consequences of diabetes/hyperglycemia were also observed. Knockdown of TXNIP in vitro protected mesangial cells grown under high-glucose conditions from apoptosis and ASK1 and p38 MAP kinase signaling [151,152]. In wild-type and TXNIP knockout mice rendered diabetic with streptozotocin, the TXNIP knockout mice showed significant protection from diabetic nephropathy. The knockout diabetic mice did not show increased thickening of the basal membrane or increased glomerular TGF-β1, collagen IV, and fibrosis was seen in the wild-type mice [153]. Loss of TXNIP provided significant protection in mice fed a HFD, showing significant protection from retinal degeneration and microvascular dysfunction due to reduced activation by the retinal endothelial cells of TXNIP/NLRP3 inflammasome [94]. TXNIP gene-deletion mice have been shown in general to be protected from the vascular consequences of high-fat/obesity or high-sugar diets compared to wild-type mice. In a model that studied the consequence of HFD and TXNIP gene deletion, it was shown that wild-type mice had significantly impaired blood flow and vascular density due to impaired angiogenesis compared to the TXNIP knockout mice. In this model, there was significantly higher IL-1β, increased numbers of infiltrating macrophages, and increased vascular endothelial growth factor (VEGF) expression and VEGF receptor activation in wild-type mice only. The enhanced vascular inflammation was due to the high-fat diet activating the TXNIP/NLRP3 inflammasome, which did not occur in TXNIP knockout mice [154].

### 6.2. Human Subjects

From the above-described experimental studies, the loss of TXNIP in mice has been beneficial in reversing the consequences of hyperglycemia but with a detrimental effect on liver fatty acid metabolism. These animals were constructed with *Txnip* gene deletions, but the loss of TXNIP has been identified in rare genetic cases in human subjects. The published case report covers children of 4, 9, and 13 years of age [155]. This showed that the loss of TXNIP is nonlethal, but the affected subjects show lactic acidosis, low-serum methionine, and impaired oxidative phosphorylation in response to glucose and pyruvate [155]. There are no reports as yet if such individuals are resistant to diabetes or neurodegenerative diseases. These studies demonstrate that the pharmacological lowering of TXNIP levels is unlikely to be associated with significant side effects.

Table 1 lists the most significant studies related to the effects of TXNIP expression in the brain or brain-derived cells. This is a subjective list based on the theme of this article and is proposed to illustrate the different features of TXNIP in interactions with the nervous system.

## 7. Modulating TXNIP Expression

A number of different classes of agents can modulate TXNIP expression, including PPAR-γ agonists, which include classes of drugs with insulin-sensitizing properties used for treating diabetes [157]. There have been conflicting results on the interactions of PPAR-γ and TXNIP. One study using human kidney proximal tubule cells showed PPAR-γ agonists attenuated high-glucose-mediated TXNIP expression [158], whereas another using human macrophages showed that the PPAR-γ agonist GW929 enhanced TXNIP expression [71]. Using a rat insulinoma cell line INS-1E to represent pancreatic beta cells, it was shown that hyperglycemia activated TXNIP expression and inhibited the activation of AMPK. Activation of AMPK with metformin or aminoimidazole-carboxamide ribonucleotide (AICAR) reduced TXNIP expression, an effect also observed when cells were treated with the lipid palmitate [142]. AMPK activation inhibited glucose-stimulated ChREBP nuclear entry and binding to the TXNIP promoter, thereby inhibiting TXNIP mRNA expression. These investigators also demonstrated that the addition of insulin to high-glucose-treated INS-1E cells reduced TXNIP expression and prevented high-glucose-mediated apoptosis. Treatment of cells with nitric oxide (NO) stimulated insulin secretion and reduced the expression of TXNIP; this effect was reversed when cells were treated with a nitric oxide synthase inhibitor [159]. A recent study showed that insulin-like growth factor-1 (IGF-1) negatively regulated TXNIP expression in vitro and in vivo. Furthermore, this study demonstrated that oxidative stress and glucose-induced TXNIP expression could be reversed by the administration of IGF-1 [160]. The calcium channel blocker verapamil, which is in widespread clinical use for hypertension, has been demonstrated to significantly inhibit TXNIP expression with therapeutic benefits. In an animal model of diabetic cardiomyopathy, three weeks of treatment with verapamil had a significant therapeutic benefit, which correlated with the reduced expression of TXNIP in cardiomyocytes [161]. The mechanism of action of verapamil appeared to be mediated by the enhanced activation of the transcription repressor nuclear factor Y (NPY) [162]. As mentioned above, verapamil was also effective in inhibiting tau phosphorylation in vivo in 5xFAD AD model mice, inhibiting Aβ-induced tau phosphorylation in vitro, and inhibiting the expression of TXNIP and the activation of p38 MAPK [123].

In a recent study, we identified a novel fluorinated derivative of curcumin that was highly effective in reducing stress-induced TXNIP expression. Cellular models examined used the human retinal pigment epithelial cell line ARPE-19, which have high constitutive levels of TXNIP expression, and macrophages derived from the human THP-1 monocyte cell line, which have lower constitutive expression. In this study, the effectiveness of fluorinated curcumin derivative Shiga Y6 compared to its non-fluorinated derivative Shiga Y5 in reducing TXNIP protein and mRNA expression under constitutive, high-glucose, endoplasmic reticulum stress, and inflammatory activation was demonstrated [163]. This compound was also effective in inducing TRX protein and mRNA levels in these cellular models. These studies demonstrate that derivatized curcumin molecules were more effective than curcumin alone in lowering TXNIP expression. Other studies have shown the effectiveness of curcumin in vitro and in vivo in inhibiting TXNIP expression but at higher doses than we used for testing the fluorinated curcumin compound [163,164]. The effect of curcumin on TXNIP expression appears to be through the activation of AMPK [81,165].

## 8. Summary

When considering the involvement of TXNIP in human acute neurological and chronic neurodegenerative diseases, there is mostly circumstantial evidence from animal and in vitro experiments about how it might accelerate pathology. It is clear there is a need for further detailed studies. With regards to AD, there have been many studies on microglia and inflammation in AD and studies showing NLRP3 inflammasome activation in AD brains without the consideration of TXNIP ([124]). TXNIP is essential for driving NLRP3 inflammasome activation and the formation of activated caspase-1 [50]. Preventing the interaction of TXNIP and NLRP3 will have significant effects. This can be done by inhibiting its expression, inhibiting the stress stimuli that causes increased expression, or manipulating the intermediate signaling pathways. As mentioned, a number of different classes of chemicals have been identified to inhibit TXNIP expression, which might have therapeutic potential for a number of diseases. The major issue for agents aimed at brain cells is the penetration of the blood–brain barrier. An issue for treating elderly patients with neurodegenerative diseases is whether inhibiting a particular target could exacerbate age-associated cancers. As decreased levels of TXNIP correlate with the increased growth of different types of cancer cells, this issue needs to be considered [166].

Understanding this protein with multiple properties related to sequestering TRX1 in the cytosol, such as sequestering TRX2 in mitochondria, modulating the expression or stability of key glucose transporters, acting as a nuclear transcription factor, and activating inflammasome, has been complicated. Its normal physiological function has not been elucidated. Some of the properties are independent of the enhancement of oxidative stress due to sequestering TRX. The interaction of oxidative stress with the activation of other properties, for example transcription factor activation, requires further study.

From this review of the literature, there is clearly a need for further studies of TXNIP expression in normal, aged, and disease-affected human brains. Only one study provided a limited description of the cellular and neuroanatomical distribution of TXNIP in human brains in AD [26]. Without this knowledge, identifying sites of TXNIP activity in relation to neuropathology, identifying the neuroanatomical distribution and cell types mediating TXNIP activity in particular, will hinder progress on developing TXNIP as a therapeutic target for brain diseases.

## Figures and Tables

**Table 1:** Summary of major studies of TXNIP in the brain or brain-derived cells.

Finding	Reference
TXNIP induced in cerebellar granular neurons by changes in potassium led to cell death	[57]
Glutamate-induced cell death in vivo and in vitro via TXNIP induced expression	[81]
TXNIP expression downregulated in vitro in neuronal cells overexpressing mitochondrial ferritin	[82]
TXNIP induction by Aβ in microglia resulting in activation inhibited by antioxidant resveratrol	[84]
High-fat-diet fed rats had increased expression of TXNIP in astrocytes, retinal endothelial cells, and microglia	[88]
Increased TXNIP expression in a rat model of subarachnoid hemorrhage in neurons, astrocytes, and microglia correlated with cell death, which was reduced by inhibiting TXNIP expression	[156]
Increased TXNIP expression in a rat model of subarachnoid hemorrhage in neurons, astrocytes, and microglia correlated with cell death which was reduced by inhibiting TXNIP expression	[156]
Downregulation of TXNIP in RPE cell line by oxidative stress reduced cell proliferation	[99]
Decreased expression of TXNIP in glioma cells correlated with poor patient outcome	[100]
TXNIP was immunolocalized to neurons and microglia in normal drosophila and rat brain tissue	[108]
Increased neuronal pyroptosis in a rat model of cerebral thrombosis mediated by elevated TXNIP expression and inflammasome activation	[117]
TXNIP overexpression in substantia nigra of mice caused the loss of dopaminergic neurons	[118]
Increased expression of TXNIP mRNA, protein, and numbers of immunopositive cells in AD brain	[26]
Increased TXNIP expression in the brain of an AD mouse model but reduced expression in the spleen	[122]
